# Characterization of Non-Specific Uptake and Retention Mechanisms of [^177^Lu]Lu-PSMA-617 in the Salivary Glands

**DOI:** 10.3390/ph16050692

**Published:** 2023-05-03

**Authors:** Nathalie Heynickx, Charlotte Segers, Amelie Coolkens, Sarah Baatout, Koen Vermeulen

**Affiliations:** 1 Nuclear Medical Applications Institute, Belgian Nuclear Research Centre (SCK CEN), 2400 Mol, Belgium; nathalie.heynickx@sckcen.be (N.H.);; 2 Department of Molecular Biotechnology, Faculty of Bioscience Engineering, Ghent University, 9000 Ghent, Belgium; 3 Department of Human Structure and Repair, Faculty of Medicine and Health Sciences, Ghent University, 9000 Ghent, Belgium

**Keywords:** PSMA targeted radionuclide therapy, prostate cancer, salivary gland toxicity

## Abstract

The radionuclide therapy [^177^Lu]Lu-PSMA-617 was recently FDA-approved for treatment of metastatic castration-resistant prostate cancer. Salivary gland toxicity is currently considered as the main dose-limiting side effect. However, its uptake and retention mechanisms in the salivary glands remain elusive. Therefore, our aim was to elucidate the uptake patterns of [^177^Lu]Lu-PSMA-617 in salivary gland tissue and cells by conducting cellular binding and autoradiography experiments. Briefly, A-253 and PC3-PIP cells, and mouse kidney and pig salivary gland tissue, were incubated with 5 nM [^177^Lu]Lu-PSMA-617 to characterize its binding. Additionally, [^177^Lu]Lu-PSMA-617 was co-incubated with monosodium glutamate, ionotropic or metabotropic glutamate receptor antagonists. Low, non-specific binding was observed in salivary gland cells and tissues. Monosodium glutamate was able to decrease [^177^Lu]Lu-PSMA-617 in PC3-PIP cells, mouse kidney and pig salivary gland tissue. Kynurenic acid (ionotropic antagonist) decreased the binding of [^177^Lu]Lu-PSMA-617 to 29.2 ± 20.6% and 63.4 ± 15.4%, respectively, with similar effects observed on tissues. (RS)-MCPG (metabotropic antagonist) was able to decrease the [^177^Lu]Lu-PSMA-617 binding on A-253 cells to 68.2 ± 16.8% and pig salivary gland tissue to 53.1 ± 36.8%. To conclude, we showed that the non-specific binding on [^177^Lu]Lu-PSMA-617 could be reduced by monosodium glutamate, kynurenic acid and (RS)-MCPG.

## 1. Introduction

At present, prostate cancer is still a major health burden with 1.4 million new cases worldwide and nearly 400,000 deaths annually [1]. At early disease stages, treatment efficacy is high, but it decreases when the disease becomes more advanced. The most advanced stage of metastatic castration-resistant prostate cancer (mCRPC) is often incurable, resulting in a five-year survival rate of merely 15% [2]. Recently, the Food and Drug Administration (FDA) approved radionuclide therapy with [^177^Lu]Lu-PSMA-617, for treatment of mCRPC in patients with a progressive disease after chemo- and androgen deprivation therapy [3].

[^177^Lu]Lu-PSMA-617, targets the prostate specific membrane antigen (PSMA), also known as glutamate carboxypeptidase II (GCP II), which is shown to be overexpressed in 90–100% of prostate cancer cases [4]. That, together with the large extracellular domain, makes PSMA a very interesting target for targeted radionuclide therapy. However, the expression of PSMA is not strictly limited to prostate cancer tissue, as PSMA expression was also observed in the vasculature of other neoplasms and healthy tissues such as the proximal tubules of the kidney and the salivary glands [5]. The latter ones are of main interest, since the salivary glands are currently considered to be the dose-limiting organs following PSMA-targeted radionuclide therapy with small molecules [6]. Human salivary glands can be subdivided into three major pairs (parotid, submandibular and sublingual) and minor salivary glands. Despite PSMA expression being much lower in the salivary glands compared to the prostate cancer tissue, salivary glands show a high uptake of PSMA-targeted small molecules, suggesting a non-specific retention mechanism [7]. The ionizing radiation delivered to the salivary glands following PSMA-targeted radionuclide therapy damages the salivary glands, resulting in xerostomia, a debilitating condition, which severely decreases the quality of life and can even result in treatment discontinuation [6]. When treating patients with PSMA-targeted small molecules combined with beta-emitting radionuclides, such as ^177^Lu, xerostomia is often reversible. However, irreversible damage has been observed using the alpha particle emitter, ^225^Ac [8].

**Figure 1:**
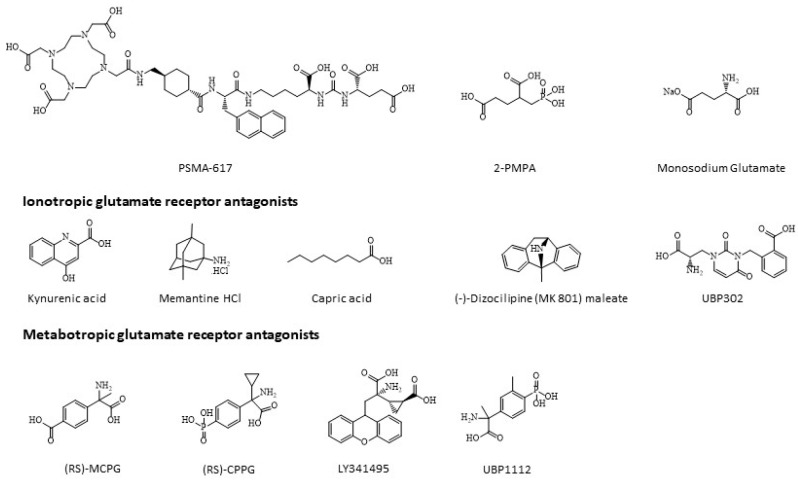
Chemical structures of compounds used in this study.

The mechanisms underlying the retention of [^177^Lu]Lu-PSMA-617 in the salivary glands remain incompletely understood. Urea-based PSMA-targeted small molecules, such as PSMA-617, target PSMA via interaction between the glutamate moiety of PSMA-targeted molecules and the enzymatic pocket of PSMA having high glutamate affinity [8,9]. With this in mind, monosodium glutamate (MSG) has been investigated as a compound to decrease retention of PSMA-targeting small molecules in the salivary glands [10,11,12]. While being successful at decreasing the healthy organ uptake of PSMA-targeting compounds, it also reduced tumor uptake, making it difficult to implement in clinical practice. However, these studies suggest that the uptake in the salivary glands of PSMA-targeted small molecules with a glutamate moiety might be reduced when interfering with the high affinity glutamate binding pocket of PSMA. Previous autoradiography studies have already indicated that the salivary glands suffer largely from non-specific accumulation of PSMA-targeted small molecules, resulting in salivary gland toxicity [13]. In this study, we describe the uptake pattern of [^177^Lu]Lu-PSMA-617 in prostate cancer and salivary gland cells. Further, autoradiography studies were conducted on pig salivary gland tissue and compared to mouse kidney tissue as a positive control. Additionally, histology was performed to characterize different pig salivary gland sections. Lastly, different glutamate receptor antagonists were used to investigate binding specificity of [^177^Lu]Lu-PSMA-617 to the salivary glands (Figure 1).

## 2. Results

### 2.1. Radiolabeling of PSMA-617 with Lutetium-177

The radiolabeling of PSMA-617 with ^177^Lu with a molar activity of 50 MBq/nmol resulted in radiochemical yields higher than 95%. The radiolabeled product, [^177^Lu]Lu-PSMA-617, was diluted in the appropriate buffer to continue further experiments. The stability of the radiolabeled product was determined once at different time points (24, 48 and 72 h) after radiolabeling in different media (radiolabeling buffer, phosphate-buffered saline (PBS), fetal bovine serum (FBS) and complete growth media (Table 1).

### 2.2. Characterization of Pig Salivary Gland Sections by Immunohistochemistry

**Figure 2:**
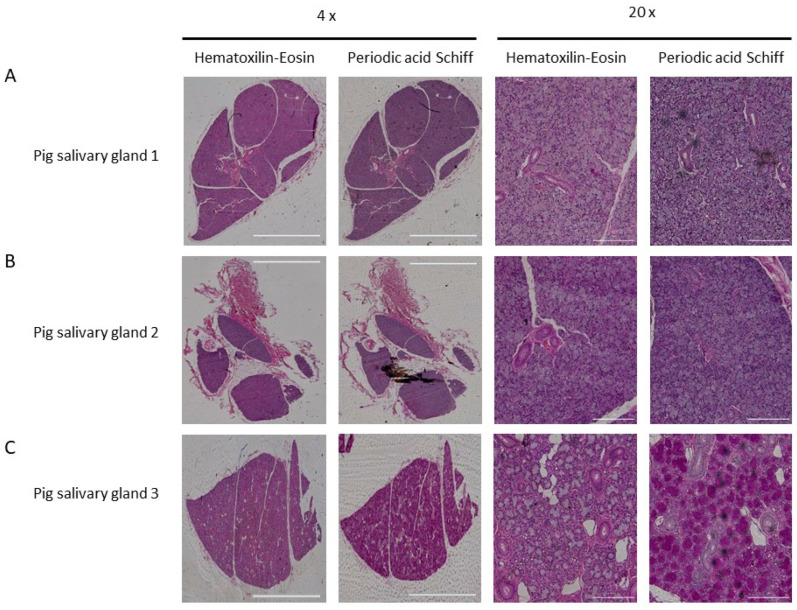
Immunohistochemistry of pig salivary gland tissue. Tissue sections from different salivary glands Panels (**A**–**C**) were stained with H&E and PAS. Images represent the full salivary gland at 4× magnification (left two images) and zoomed in to acinar structures at 20× magnification (right two images). The scale bars represent 2000 µm for the 4× magnification and 200 µm for the 20× magnification.

To study the general tissue morphology, a hematoxylin and eosin (H&E) staining was conducted on different paraffinized pig salivary gland tissues. To specifically stain mucous acinar structures, periodic acid–Schiff (PAS) staining was performed. Both pig salivary glands 1 and 2 showed dense acinar morphology, while staining negative for mucous acini (Figure 2A,B). In contrast, salivary gland 3 stained positive for mucous acini (Figure 2C).

### 2.3. Characterization of [^177^Lu]Lu-PSMA-617 Binding and Internalization in Salivary Gland Cells

**Figure 3:**
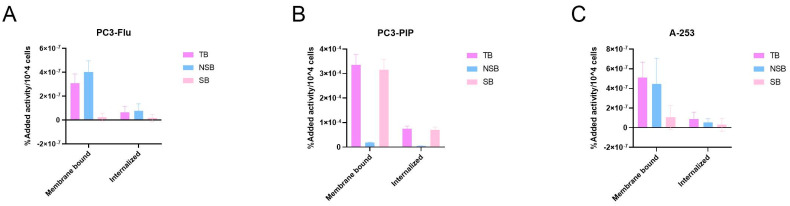
[^177^Lu]Lu-PSMA-617 binding and internalization graphs for PC3-Flu (**A**), PC3-PIP (**B**) and A-253 (**C**) cells. Data were depicted as percentage added activity per 10^4^ cells (%AA/10^4^ cells). Error bars indicate the standard deviation. TB = total binding; NSB = non-specific binding; SB = specific binding.

Cellular binding and internalization of [^177^Lu]Lu-PSMA-617 was investigated using PC3-PIP and A-253 cells using the potent PSMA-inhibitor 2-(Phosphonomethyl)pentanedioic acid (2-PMPA) to block specific binding to the PSMA receptor. Low specific [^177^Lu]Lu-PSMA-617 binding was observed on the PC3-Flu cells (3.08 ± 0.79 × 10^−7^%AA/10^4^ cells), with the majority of the radioactivity detected on the membrane. (Figure 3A). On PSMA-positive PC3-PIP cells, the observed binding (3.36 ± 0.42 × 10^−4^%AA/10^4^ cells) was highly specific as demonstrated by the co-incubation with 2-PMPA (Figure 3B). On A-253 salivary gland cells, the binding of [^177^Lu]Lu-PSMA-617 was restricted to 5.12 ± 1.57 × 10^−7^%AA/10^4^ cells. Despite the majority of this binding being non-specific, a minor amount of this appears to be specific (1.06 ± 1.22 × 10^−7^%AA/10^4^ cells) (Figure 3C). On all cell types, the majority of [^177^Lu]Lu-PSMA-617 was present in the membrane bound fraction, in line with the antagonistic nature of PSMA-617.

### 2.4. Characterization of [^177^Lu]Lu-PSMA-617 Binding on Salivary Gland Tissue

In vitro autoradiography experiments were conducted on the mouse kidney (PSMA-expressing positive control tissue) and the different pig salivary gland tissues. Saturation binding experiments were performed on all tissues. Again, 2-PMPA was used to determine non-specific binding.

**Figure 4:**
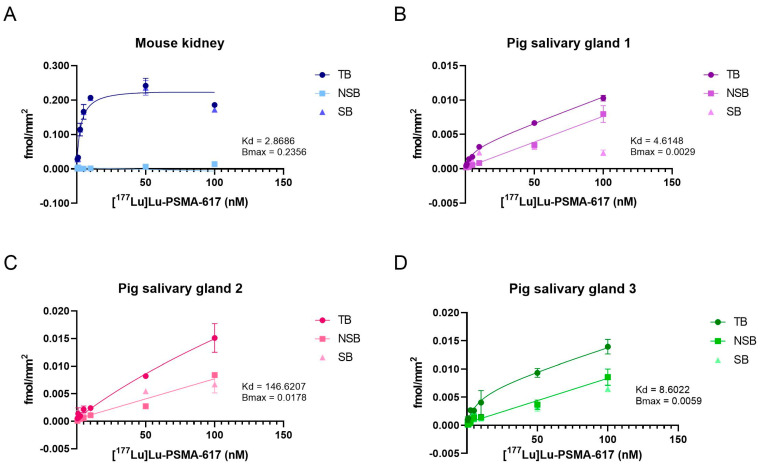
Saturation binding curves of the mouse kidney (**A**), pig salivary gland 1 (**B**), 2 (**C**) and 3 (**D**) tissues. Binding affinity (Kd in nM) and PSMA receptor density (Bmax in fmol/mm^2^) are indicated for each tissue. TB = total binding; NSB = non-specific binding; SB = specific binding.

The binding of [^177^Lu]Lu-PSMA-617 to the mouse kidney tissue was nearly completely specific and expressed high affinity (Kd = 2.9 nM, (Figure 4A, Table 1). The uptake of [^177^Lu]Lu-PSMA-617 in three different pig salivary gland sections was partially specific, indicated by the total binding curves in Figure 4B–D. At low [^177^Lu]Lu-PSMA-617 concentrations (until 10 nM), the binding to salivary gland sections showed a specific component, while treatment with higher concentrations appeared to induce a non-specific binding. Pig salivary glands 1 and 3 showed similar affinity values, in contrast to pig salivary gland 2 (Table 2). The PSMA receptor density (Bmax, Table 1) of the mouse kidney tissue was roughly 100-fold higher compared to pig salivary glands 1 and 3. Pig salivary gland 2 expressed the lowest affinity and more pronounced non-specific binding, but a higher Bmax. The calculated binding potentials (BP) of the different tissues again roughly show a 100-fold difference between the mouse kidney tissue and salivary glands 1 and 3, which were very similar. Salivary gland 2 showed the least amount of binding of [^177^Lu]Lu-PSMA-617, indicating the lowest amount of PSMA receptors present.

### 2.5. Cytotoxicity of Monosodium Glutamate, Ionotropic and Metabotropic Glutamate Receptor Antagonists

To validate whether the proposed concentrations of the compounds to perform competition studies (Table 2) were safe to use on PC3-PIP and A-253 cell lines, the cytotoxicity of the respective compounds was investigated using a sulforhodamine B (SRB) assay.

**Figure 5:**
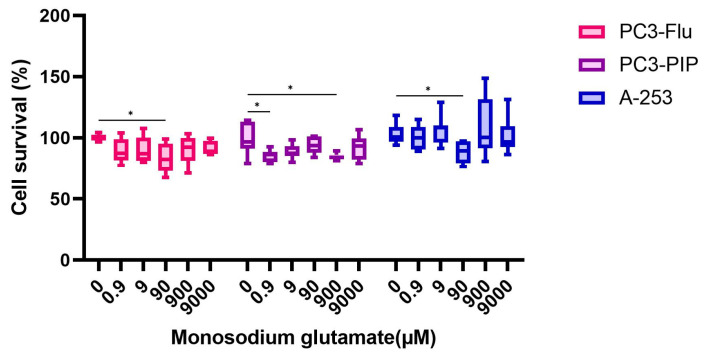
Cytotoxicity data for MSG. Data were normalized to the untreated control per cell type. Data were represented as mean cell survival percentage (%) ± standard deviation. * Indicates a *p*-value < 0.05 for comparison respective to the untreated control for each respective cell type.

Treatment with MSG showed varying effects on cell survival (Figure 5). For PC3-Flu cells, only 90 µM of MSG significantly decreased cell survival to 84.2 ± 11.4%. On PC3-PIP cells, 0.9 and 900 µM MSG had a similar effect, decreasing cell survival to 85.3 ± 4.7% and 84.6 ± 2.5%, respectively. On A-253 cells, 90 µM MSG decreased cell survival to 88.2 ± 8.6%. As cell survival did not drop below 50%, the tested concentrations were further used in the competition studies.

**Figure 6:**
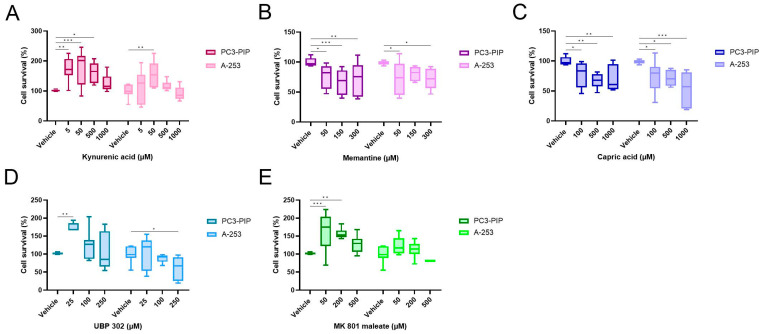
Cytotoxicity data of the ionotropic glutamate receptor antagonists kynurenic acid (**A**), memantine (**B**), capric acid (**C**); UBP302 (**D**) and MK 801 maleate (**E**). Data were normalized to the vehicle condition per compound and per cell type. Data were represented as mean cell survival percentage (%) ± standard deviation. * Indicates a *p*-value < 0.05; ** indicates a *p*-value < 0.01 and *** indicates a *p*-value < 0.001 for comparisons in respect to the vehicle condition per cell type.

Treatment with up to 1000 µM kynurenic acid showed no significant decrease in cell survival of PC3-PIP and A-253 cells (Figure 6A). Treatment with memantine (50 to 300 µM) and capric acid (100 to 1000 µM) showed a significant decrease in cell survival (Figure 6B,C). The highest concentration of UBP302 showed a decrease in A-253 cell survival, but not in PC3-PIP cell survival (Figure 6D). Treatment with MK 801 maleate induced no significant decrease in cell survival (Figure 6E). Despite some compounds decreasing cell survival, mean cell viability remained above 50% for all tested concentrations.

**Figure 7:**
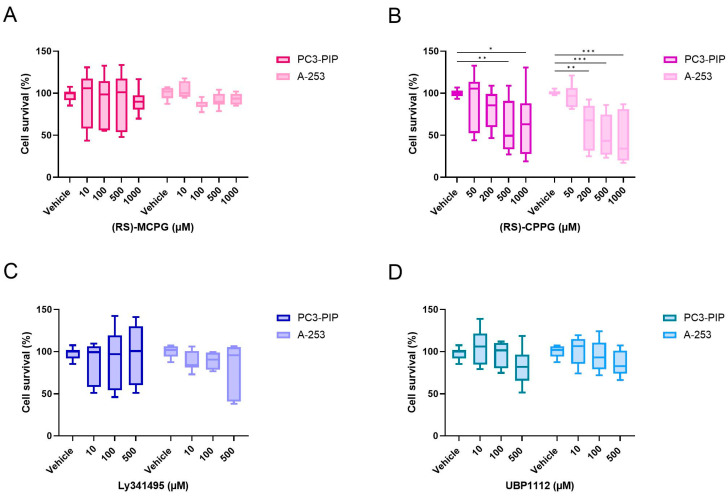
Cytotoxicity data of the metabotropic glutamate receptor antagonists (RS)-MCPG (**A**), (RS)-CPPG (**B**), Ly341495 (**C**) and UBP1112 (**D**). Data were normalized to the vehicle condition per compound and per cell type. Data were represented as mean cell survival percentage (%) ± standard deviation. * Indicates a *p*-value < 0.05; ** indicates a *p*-value < 0.01 and *** indicates a *p*-value < 0.001 for comparisons in respect to the vehicle condition per cell type.

When testing the metabotropic glutamate receptor antagonists, only (RS)-CPPG showed cytotoxicity starting at a concentration of 200 µM, which further increased at 1000 µM. This effect was more pronounced in A-253 cells compared to PC3-PIP cells (Figure 7B). The other metabotropic glutamate receptor antagonists (RS)-MCPG, Ly341495 and UBP1112 showed no significant effects on cell survival in both PC3-PIP and A-253 cells (Figure 7A,C,D).

### 2.6. Competition Study with Monosodium Glutamate

We investigated whether the co-incubation of [^177^Lu]Lu-PSMA-617 with MSG would be able to decrease the [^177^Lu]Lu-PSMA-617 uptake through the blocking of non-specific, saturable binding sites.

**Figure 8:**
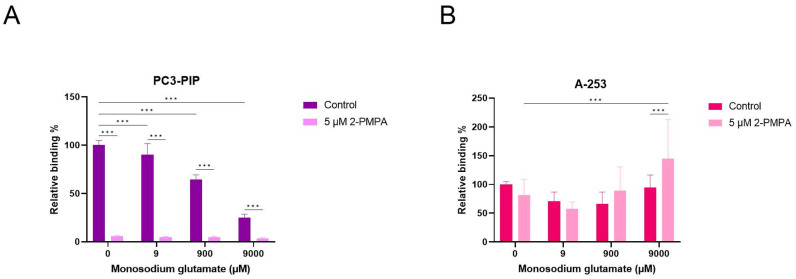
Competition study with MSG in PSMA-positive PC3-PIP prostate cancer (**A**) and A-253 salivary gland (**B**) cells. Cells were incubated with [^177^Lu]Lu-PSMA-617 (5 nM) and 2-PMPA (5 µM) and/or increasing concentrations of MSG (0 to 9000 µM). Data were normalized to the untreated control condition and represented as mean ± standard deviation. *** indicates a *p*-value < 0.001.

The PSMA-positive PC3-PIP and A-253 salivary gland cells were treated with [^177^Lu]Lu-PSMA-617 (5 nM) and increasing concentrations of (0 to 9000 µM) MSG or 5 µM 2-PMPA as a positive control. In PC3-PIP cells, treatment with 2-PMPA resulted in a significant decrease in [^177^Lu]Lu-PSMA-617 binding to 4.68 ± 0.98%. Treatment with increasing concentrations of MSG also significantly decreased the binding of [^177^Lu]Lu-PSMA-617. At 9000 µM MSG, binding of [^177^Lu]Lu-PSMA-617 was reduced to 24.95 ± 3.64% (Figure 8A). In A-253 salivary gland cells, results were inconclusive, due to the low amount of uptake observed in these cells (Figure 8B).

**Figure 9:**
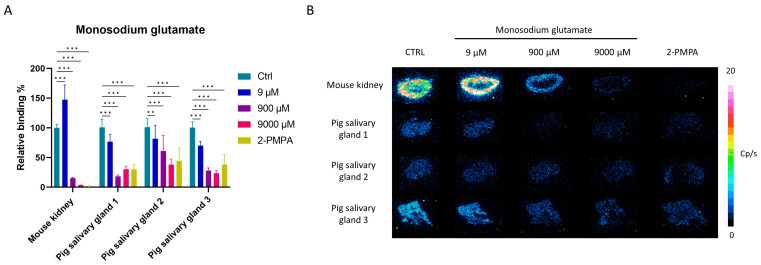
MSG competition study on the mouse kidney tissue and different pig salivary gland sections. Cryosections were treated with [^177^Lu]Lu-PSMA-617 (5 nM) and co-incubated with increasing concentrations (0 to 9000 µM) of MSG or 100 µM 2-PMPA. Panel A shows the mean relative binding percentage per tissue and treatment. Data were normalized to the untreated control condition and presented as mean relative binding percentage ± SD. ** indicates a *p*-value < 0.01 and *** indicates a *p*-value < 0.001. Panel B shows the corresponding In vitro autoradiography images.

Competition studies on the mouse kidney and pig salivary gland tissue were similarly conducted as described above. Uptake was highest in the kidney tissue, restricted to the cortex, followed by pig salivary gland 3. The uptake of [^177^Lu]Lu-PSMA-617 on pig salivary glands 1 and 2 was more limited, but saturable. On all tissues, the co-incubation of [^177^Lu]Lu-PSMA-617 (5 nM) with MSG (0 to 9000 µM) decreased binding in a concentration-dependent manner (Figure 9). Treatment with 9000 µM MSG decreased the [^177^Lu]Lu-PSMA-617 binding to the same extent as the treatment with 100 µM 2-PMPA.

### 2.7. Competition Study with Ionotropic Glutamate Receptor Antagonists

Since MSG was shown to decrease the binding of [^177^Lu]Lu-PSMA-617 to salivary glands, we investigated whether specific blocking of ionotropic glutamate receptors with different antagonists targeting different receptor subclasses was able to decrease the [^177^Lu]Lu-PSMA-617 binding on salivary glands.

**Figure 10:**
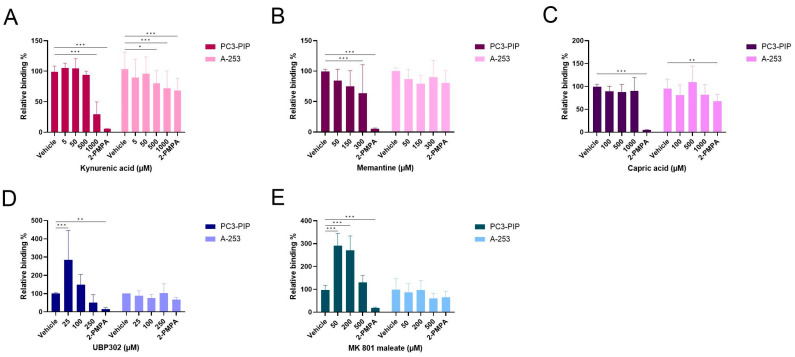
Competition study with different ionotropic glutamate receptor antagonists on PC3-PIP and A-253 cells. Cells were incubated with [^177^Lu]Lu-PSMA-617 (5 nM) and different concentrations of kynurenic acid (0 to 1000 µM) (**A**), memantine (0 to 300 µM) (**B**), capric acid (0 to 1000 µM) (**C**), UBP302 (0 to 250 µM) (**D**) and MK 801 maleate (0 to 500 µM) (**E**). Data were normalized to the control condition per compound and cell type and presented as mean relative binding ± standard deviation. * Indicates a *p*-value < 0.05; ** indicates a *p*-value < 0.01 and *** indicates a *p*-value < 0.001 for comparison with the vehicle condition per cell type.

At the cellular level, the highest concentration (1000 µM) of the pan-specific, ionotropic glutamate receptor antagonist kynurenic acid (Figure 10A) was able to reduce the [^177^Lu]Lu-PSMA-617 binding to 29.2 ± 20.6% on PC3-PIP cells and to 63.4 ± 15.4% on A-253 cells. Memantine, a N-methyl-D-aspartate (NMDA) receptor antagonist, decreased binding to 63.4 ± 46.9% on PC3-PIP cells at 300 µM, and to 79.4 ± 14.0% on A-253 cells at 150 µM (Figure 10B). The α-amino-3-hydroxy-5-methyl-4-isoxazolepropionic acid (AMPA) receptor antagonist, capric acid (Figure 10C); the kainate receptor antagonist, UBP302 (Figure 10D) and the NMDA receptor inhibitor, MK 801 maleate (Figure 10E), had no significant effect on the [^177^Lu]Lu-PSMA-617 binding on either PC3-PIP or A-253 cells.

**Figure 11:**
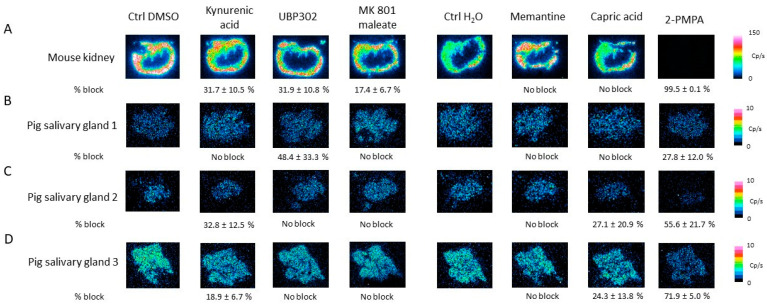
Summary of the In vitro autoradiography results of the competition study. Mouse kidney (**A**) and pig salivary gland (**B**–**D**) issues were treated with [^177^Lu]Lu-PSMA-617 (5 nM) and different concentrations of ionotropic glutamate receptor antagonists: 1000 µM kynurenic acid, 250 µM UBP302, 500 µM MK 801 maleate, 300 µM memantine and 1000 µM capric acid or 2-PMPA (100 µM). Percentage block (% block) is indicated underneath the autoradiography images and is represented as mean ± standard deviation.

Next, In vitro autoradiography on the mouse kidney and pig salivary gland tissue was performed by treating the tissues with [^177^Lu]Lu-PSMA-617 (5 nM) and different concentrations of ionotropic glutamate receptor antagonists. On the mouse kidney tissue (Figure 11A), 1000 µM kynurenic acid (31.7 ± 10.5%), 250 µM UBP302 (31.9 ± 10.8%) and 500 µM MK 801 maleate (17.4 ± 6.7%) were able to reduce the [^177^Lu]Lu-PSMA-617 binding, while memantine and capric acid showed no effect. On pig salivary gland 1 (Figure 11B), only UBP302 (48.8 ± 33.3%) induced a decrease in [^177^Lu]Lu-PSMA-617 binding. A decrease in the binding of [^177^Lu]Lu-PSMA-617 was observed on pig salivary gland 2 and 3 (Figure 11C,D), by co-incubation with kynurenic acid (32.8 ± 12.5 and 18.9 ± 6.7%) and capric acid (27.1 ± 20.9 and 24.3 ± 13.8%).

### 2.8. Competition Study with Metabotropic Glutamate Receptor Antagonists

Similar to the competition study with ionotropic glutamate receptor antagonists, we investigated whether the non-specific binding of [^177^Lu]Lu-PSMA-617 on salivary glands could be attributed to the binding to different subclasses of metabotropic glutamate receptors.

**Figure 12:**
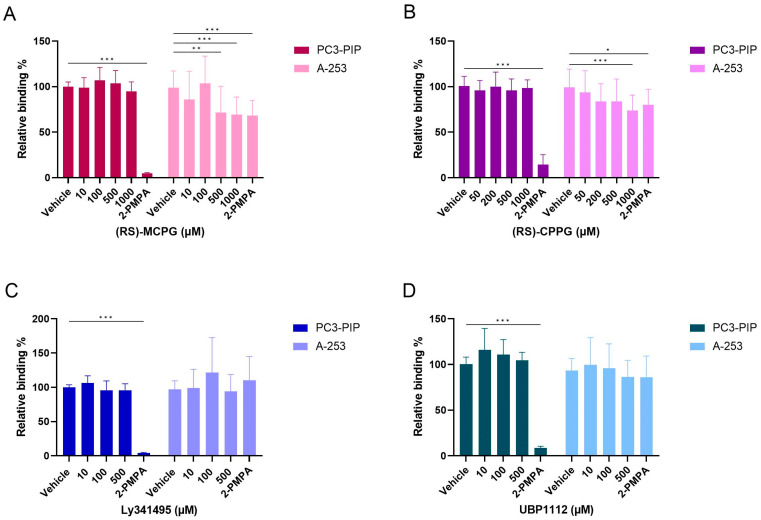
Competition study with different metabotropic glutamate receptor antagonists. Cells were treated with [^177^Lu]Lu-PSMA-617 (5 nM) and different concentrations of metabotropic glutamate receptor antagonists: (RS)-MCPG (0 to 1000 µM) (**A**), (RS)-CPPG (0 to 1000 µM) (**B**), Ly341495 (0 to 500) (**C**) and UBP1112 (0 to 500 µM) (**D**) or 2-PMPA (5 µM). Data were normalized per compound and cell type to the control condition and presented as a mean relative binding percentage ± standard deviation. * Indicates a *p*-value < 0.05; ** indicates a *p*-value < 0.01 and *** indicates a *p*-value < 0.001 in comparison with the vehicle condition per cell type.

At the cellular level, none of the investigated compounds reduced the binding of [^177^Lu]Lu-PSMA-617 (5 nM) on PC3-PIP prostate cancer cells (Figure 12). On A-253 salivary gland cells, 500 and 1000 µM of (RS)-MCPG reduced the binding of [^177^Lu]Lu-PSMA-617 to 71.7 ± 28.7% and 68.2 ± 16.8%, respectively (Figure 12A). Similarly, co-incubation with 1000 µM of (RS)-CPPG reduced the [^177^Lu]Lu-PSMA-617 binding on A-253 cells to 73.7 ± 17.0% (Figure 12B). The other tested compounds Ly341495 and UBP1112 had no significant effect on the [^177^Lu]Lu-PSMA-617 binding (Figure 12C,D).

**Figure 13:**
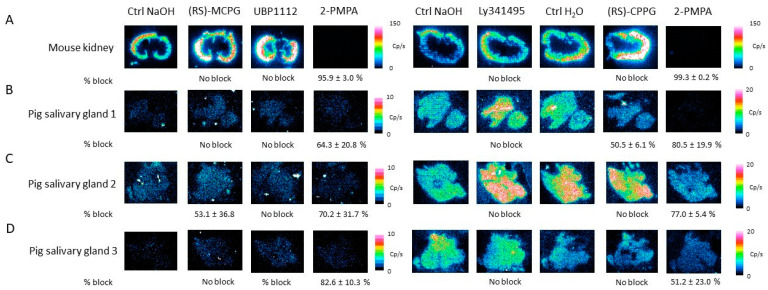
Summary of the In vitro autoradiography results using metabotropic glutamate receptor antagonists on the mouse kidney (**A**) and pig salivary gland tissues (**B**–**D**). Tissues were treated with [^177^Lu]Lu-PSMA-617 (5 nM) and different concentrations of metabotropic glutamate receptor antagonists: 1000 µM (RS)-MCPG, 1000 µM (RS)-CPPG, 500 µM Ly341495 and 500 µM UBP1112 or 2-PMPA (100 µM). Percentage block (% block) is indicated underneath the respective autoradiography images and represented as a mean ± standard deviation. Results are shown on the left panel for (RS)-MCPG and UBP1112 (*n* = 3), and Ly341495 and (RS)-CPPG on the right (*n* = 2).

Similarly, the mouse kidney and pig salivary gland tissues were treated with [^177^Lu]Lu-PSMA-617 (5 nm) and different metabotropic glutamate receptor antagonists. On the mouse kidney tissue, none of the investigated metabotropic glutamate receptor antagonists reduced the [^177^Lu]Lu-PSMA-617 binding (Figure 13A). On the pig salivary gland tissue (Figure 13B–D), only 1000 µM (RS)-MCPG (53.1 ± 36.8%) and 1000 µM (RS)-CPPG (50.5 ± 6.1%) showed any effect on the [^177^Lu]Lu-PSMA-617 binding although this effect was only seen on pig salivary glands 1 and 2.

## 3. Discussion

Targeted radionuclide therapy using PSMA-targeted small molecules has emerged as an efficient new treatment option for patients with metastatic castration-resistant prostate cancer. Recently, this resulted in the FDA approval of [^177^Lu]Lu-PSMA-617 as an end-stage treatment of this patient population. However, clinical results already indicated a significant salivary gland uptake of PSMA-targeted small molecules [3]. At present, the salivary glands are considered to be the dose-limiting organ of treatment with PSMA-targeted small molecules, but mechanisms underlying the uptake and retention in these glands remain largely unknown [6]. Therefore, our aim was to elucidate mechanisms of the uptake and retention of the well-known compound [^177^Lu]Lu-PSMA-617 In vitro on cells by competition studies and on tissues by In vitro autoradiography experiments.

PSMA, also known as GCP II or folate hydrolase I, is an interesting target for prostate cancer therapy due to the overexpression on prostate cancer cells. However, this PSMA expression is not strictly limited to prostate cancer tissue, as PSMA expression has also been reported in the kidneys, small intestines and salivary glands, contributing to healthy organ toxicity. For the salivary glands, PSMA expression was investigated by quantitative polymerase chain reaction (qPCR), Western blots and immunohistochemistry and was reported to be rather low and restricted to specific subsites [7]. Previous dosimetric studies also showed that the uptake of PSMA-targeted small molecules differed between the different salivary glands. The highest uptake (defined as the standardized uptake value, SUV) was observed in the submandibular gland (SUV_max_ 1 h post injection = 14.5), followed by the parotid gland (SUV_max_ 1 h post injection = 13.8) and lastly the sublingual gland (SUV_max_ 1 h post injection = 6.1) [14,15]. The parotid gland was reported to have the highest variation in SUV_max_, which could explain the large difference in BP between salivary glands 1 and 2 observed in our study, both having parotid gland morphology. In contrast, salivary gland 3 had the highest BP amongst the salivary gland sections and our immunohistochemistry data showed mixed serous and mucous acini, being indicative of submandibular gland morphology and uptake pattern [16,17]. The binding of [^177^Lu]Lu-PSMA-617 to the pig salivary gland was heterogeneous and confined to glandular areas as observed in the In vitro autoradiography study and H&E stainings. This finding was in line with previous reports [15]. The glandular areas contain the salivary gland acinar structures responsible for saliva secretion. More so, PSMA glands are expressed in the acinar glandular cells and not in ductal cells [15]. The higher [^177^Lu]Lu-PSMA-617 retention in the glandular areas and subsequent damage to acinar structures might thus relate to clinically observed xerostomia and salivary gland dysfunction.

The low PSMA expression on salivary glands indicates that the high uptake observed in PSMA-PET/CT scans in patients treated with PSMA-targeted small molecules is at least in part non-specific [18]. This hypothesis was validated in our research where at the cellular level, there was a high, specific binding of [^177^Lu]Lu-PSMA-617 on PSMA positive PC3-PIP cells, while the binding on A-253 cells was low and mainly non-specific. We also performed saturation binding studies on mouse kidney tissue and different sections of pig salivary glands. These results validated the binding profile found on cells, where the mouse kidney tissue shows a high specific binding while the pig salivary glands showed mainly non-specific binding. The Bmax and Kd values we found for the tested pig salivary gland tissues are in line with previous research [13].

Looking into the structure of the PSMA receptor, it consists of a large extracellular domain which can be divided into three separate domains, including the protease, apical and C-terminal domain. The enzymatic function of PSMA includes the hydrolysis of terminal glutaminyl residues of *N*-acetyl aspartyl glutamate or folates, but its exact function in the salivary glands remains elusive [19]. The majority of PSMA-targeted small molecules under investigation today are urea-based inhibitors consisting of a glutamate-urea-lysine moiety designed for PSMA targeting [20]. In this regard, MSG was investigated preclinically for its potential ability to reduce binding of PSMA-targeted small molecules to PSMA in the salivary glands. Previous research already showed that MSG was indeed able to reduce the uptake of [^68^Ga]Ga-PSMA-11 in salivary glands, while maintaining the tumor uptake. Therefore, it was suggested that MSG reduced the binding to PSMA by competing with off-target binding sites in healthy tissues such as kidneys and salivary glands [11]. In our research, we confirmed that the co-incubation of [^177^Lu]Lu-PSMA-617 with MSG indeed decreased the binding on salivary gland tissues. However, we also observed a decreased binding of [^177^Lu]Lu-PSMA-617 when treated with higher MSG concentrations, suggesting that MSG might be able to reduce tumor uptake when dosing is too high. This data is in accordance with published reports, where decreased uptake was observed in patients treated with [^68^Ga]Ga-PSMA-11 after the oral administration of MSG [21].

We also investigated the ability of ionotropic and metabotropic glutamate receptor antagonists to reduce the binding of [^177^Lu]Lu-PSMA-617 on prostate and salivary gland cells as well as mouse kidney and pig salivary gland tissues. We showed that both on cells and tissues, the ionotropic glutamate receptor antagonist kynurenic acid and the metabotropic glutamate receptor antagonists (RS)-MCPG and (RS)-CPPG had a decreasing effect on the [^177^Lu]Lu-PSMA-617 binding. However, for (RS)-CPPG, the cytotoxicity assays showed a decreased cell survival for all (RS)-CPPG concentrations except 50 µM. Therefore, the observed reduced binding might be attributed to a lower number of cells present in these conditions.

Most PSMA-targeted small molecules today utilize a glutamyl residue to bind to the active site of PSMA. Therefore, an alternative suggested mechanism for off-target binding of PSMA-targeted small molecules in the salivary glands involves the anion/H^+^ transporter SLC17A5 (sialin). Sialin is highly expressed in salivary glands and, when present in the plasma membrane of salivary gland cells, it can mediate the electrogenic co-transport of anions such as aspartate and glutamate [22]. This anion co-transporter expressed in the salivary glands with an affinity for glutamate might be a reason for the off-target binding related to the glutamyl residues in PSMA-targeted small molecules, and for the effect of MSG and specific ionotropic and metabotropic glutamate receptor antagonists on the binding and retention of these small molecules in the salivary glands. The hypothesis of off-target accumulation of PSMA-targeted small molecules is further supported by the fact that antibodies designed to target PSMA, such as the monoclonal antibody J591, show no uptake in the salivary glands [23].

One of the functions of PSMA encompasses the modulation of glutamate signaling via the metabotropic glutamate receptor pathway, resulting in the cleavage of glutamate from dietary folic acids and the neurotransmitter *N*-acetyl-L-aspartyl-L-glutamate. This function could result in the affinity of glutamate-urea-lysine moieties for other PSMA-like proteins such as metabotropic glutamate receptors. A computational study suggested that PSMA-targeted molecules with a glutamate-urea-lysine moiety can indeed bind to metabotropic glutamate receptors and *N*-acetyl-L-aspartyl-L-glutamate [24]. In our study, we utilized several metabotropic glutamate receptor antagonists and found that only (RS)-MCPG (a group I and II metabotropic glutamate receptor antagonist) had an effect on [^177^Lu]Lu-PSMA-617 binding. However, our study was limited to three or four concentrations of each of the antagonists tested, so it might be warranted to screen more metabotropic glutamate receptor antagonists with more variety in tested concentrations.

Another suggested mechanism for off-target accumulation of PSMA-targeted small molecules is cross-reactivity with glutamate carboxypeptidase III, which is highly expressed in both kidneys and salivary glands. Additionally, it shows a high degree of homology with the PSMA receptor [25]. To further elucidate the exact mechanism of off-target accumulation of PSMA-targeted small molecules, it might be interesting to repeat similar experiments to those we performed with inhibitors of sialin receptors and glutamate carboxypeptidase III.

A major limitation of this study comprises the use of A-253 cells to investigate the binding of [^177^Lu]Lu-PSMA-617 to salivary glands. First, these cells are derived from a carcinoma of the submandibular gland, although literature has described these cells as having maintained many healthy tissue traits, including aquaporin expression important for salivary gland function [26]. However, it remains less than adequate to study healthy tissue characteristics on cancerous tissue models. But the fact is, there is a lack of proper healthy salivary gland cell models that are readily available. On top of this, studying salivary gland uptake in vivo also remains challenging because mice show a higher kidney PSMA expression profile compared to humans, acting as a sink for circulating [^177^Lu]Lu-PSMA-617 [27]. However, we used this characteristic and included mouse kidney tissue as a positive control in the autoradiography studies. Pig salivary glands are described to be close human homologues regarding salivary gland structure and function. Furthermore, pig PSMA shows a 91% sequence homology to the human PSMA protein [28,29]. Western blot and immunohistochemistry on several pig tissues showed similar expression profiles of PSMA compared to human tissue. More so, the ratio of PSMA expression between pig salivary glands and prostate cancer cells is also similar compared to humans as pig salivary glands are reported to have a 500-fold decrease in PSMA expression compared to prostate cancer cells [13,30]. However, a complete in vivo evaluation on more relevant animal models with higher homology in the PSMA expression pattern to human salivary glands, such as pigs, remains difficult from an ethical point of view [23,26].

A second limitation of using the A-253 cells is the very low uptake of [^177^Lu]Lu-PSMA-617, as indicated by our binding and internalization studies. The lack of proper cellular models hampers conducting mechanistic studies on why PSMA-targeted small molecules are binding to, and retained in, the salivary glands. This highlights the need for better models to investigate salivary gland uptake of PSMA-targeted compounds, which better represent the uptake pattern as seen in humans, facilitating the development of appropriate countermeasures.

## 4. Materials and Methods

### 4.1. Chemicals

PSMA-617 (vipivotide tetraxetan, HY-117410), 2-PMPA (HY-100788) were purchased from MedchemExpress (Monmouth Junction, NJ, USA). The sulforhodamine B (SRB) based In vitro toxicology assay kit (TOX6) and L-glutamic acid monosodium salt hydrate (monosodium glutamate, MSG, G5889) were purchased from Sigma-Aldrich (Overijse, Belgium). Kynurenic acid (S4719), memantine HCl (S2043), capric acid (S6906) and (-)-Dizocilpine (MK 801) maleate (S2857) were purchased from SelleckChem (Planegg, Germany). UBP 302 (2079) was purchased from Tocris Biosciences (Abingdon, UK). (RS)-MCPG (sc-202325), (RS)-CPPG (sc-203448), LY341495 (sc-361244A) and UBP1112 (sc-204368) were purchased from Santa Cruz Biotechnologies (Heidelberg, Germany). Compounds were dissolved either in H_2_O, 0.05 M NaOH or DMSO.

### 4.2. Cell Culture

PC3-Flu (PSMA-negative) and PC3-PIP (PSMA-positive) prostate cancer cells (kindly provided by Dr. Pomper, John Hopkins University, Baltimore, MD, USA) were grown in RPMI-1640 High glucose Low sodium bicarbonate medium (Gibco™, Thermo Fischer Scientific, A1049101, Geel, Belgium) supplemented with 10% FBS, 100 µmL penicillin-streptomycin and 2 µg/mL Puromycin [12]. A-253/HTB-41 submandibular salivary gland cells were purchased from the American Type Culture collection (ATCC, Manassas, VA, USA) and cultured using McCoy’s 5A medium (ATCC, 30-2007) supplemented with 10% FBS and 100 u/mL penicillin/streptomycin. All cell lines were maintained in a humidified 37 °C incubator with 5% CO_2_ and sub-cultivated when at 80–90% confluency.

### 4.3. In Vitro Tissue Models

Healthy mouse kidneys were dissected from BALB/c mice. The mice were sacrificed by an overdose pentobarbital (200 µL of 60 mg/mL) after which the kidneys were dissected and rinsed with saline to remove blood. Pig salivary glands were obtained in collaboration with the Medanex clinic (Diest, Belgium). After dissection, tissues of interest were either prepared for snapfreezing or paraffin embedding. For snapfreezing, tissues were embedded in Tissue Tek (Tissue-Tek O.C.T., Sakura Finetek Europe B.V, Alphen aan den Rijn, the Netherlands) and snapfrozen using 2-methylbutane at −40 °C. Tissues were stored at −20 °C until further processing. For paraffin embedding, tissues were fixed using 4% PFA and stored in 70% ethanol until further use. Next, tissues were dehydrated in an ethanol/xylol series and submerged in paraffin. After hardening, tissues were stored until further use. Cryosections of 20 µm thickness were sliced using a cryotome (Cryostar NX50, Thermo Fisher Scientific, Geel, Belgium) and tissue sections were mounted on Superfrost Plus microscope slides. Slides were then stored at −20 °C until further use. Paraffin sections were sliced using a microtome at 7 µm thickness and mounted on microscope slides. Sections were stored at room temperature until further use.

### 4.4. Radiolabeling of PSMA-617 with Lutetium-177

[^177^Lu]LuCl_3_ was purchased from ITG (Munich, Germany). The radiolabeling with DOTA-PSMA-617 was performed as described previously [31]. In brief, DOTA-PSMA-617 was labeled with ^177^Lu at a molar activity of 50 MBq/nmol in 0.5 M sodium acetate, pH 5. The reaction mixture was placed in a Thermomixer C (Eppendorf, Hamburg, Germany) to shake (500 rpm) at 95 °C for 10 min.

The radiolabeling yield was determined using thin-layer chromatography. After cooling down, 2 µL of the reaction mixture was spotted on a glass microfiber chromatography paper strip impregnated with silica gel (iTLC-SG, Agilent Technologies, Diegem, Belgium). The elution was performed using a 0.5 M (pH 5.5) citrate solution after which the TLC papers were cut in half. Next, the activity of the top and bottom parts was counted using a 2480 WIZARD^2^ automatic y-counter (Perkin-Elmer, Mechelen, Belgium). Radiolabeling efficiency was calculated as: Radiolabeling yield = CPM_Bottom_/(CPM_Top_ + CPM_Bottom_). Reactions with a radiochemical yield >95% were diluted in an appropriate buffer for further experiments. To test the stability of the radiolabeled product, [^177^Lu]Lu-PSMA-617 was diluted in several relevant media and tested at various time points after radiolabeling until 72 h using iTLC as described above.

### 4.5. Cell Binding and Internalization Assays

PC3-Flu, PC3-PIP and A-253 cells were seeded at 50,000 cells/well in a 24-well plate and incubated at 37 °C and 5% CO_2_ in order for the cells to adhere to the surface. Cells were then treated with 5 nM [^177^Lu]Lu-PSMA-617 and 5 µM of the highly potent PSMA-inhibitor 2-PMPA or vehicle to distinguish between specific and non-specific binding. After 4 h of incubation at 37 °C and 5% CO_2_, cells were washed twice with PBS (corresponding to the wash fraction). Next, cells were incubated for 10 min at room temperature and washed twice with 50 mM glycine and 100 mM NaCl (pH 2.8) to collect the membrane bound fraction [26]. Lastly, cells were incubated with 1 M NaOH for 30 min and washed twice to collect the internalized fraction. Then, the activity of the fractions was measured using a 2480 WIZARD^2^ automatic y-counter. Activity measurements were converted to % added activity (%AA) and normalized for cell counts, performed by an automated MOXI Z cell counter, per cell type.

### 4.6. Tissue Saturation Binding Assay

The dissociation constant (Kd), the maximum density of receptors (Bmax) and the binding potential (ratio of Bmax over Kd) of mouse kidneys and pig salivary glands were determined using In vitro autoradiography. The autoradiography experiments were performed according to a published protocol [13]. In brief, tissue sections were air dried before incubation with 170 mM Tris-HCl and 5 mM MgCl_2_ (pH 7.4) for 10 min at room temperature. The slides were then incubated with increasing concentrations (10.5–1200 nM) of [^177^Lu]Lu-PSMA-617 in 170 mM Tris-HCl, 5 mM MgCl_2_ (pH 7.4) and 1% bovine serum albumin (BSA). In order to distinguish specific binding from non-specific binding, tissue sections were co-incubated with 100 µM 2-PMPA. After 1 h of incubation, the treatment was removed from the slides and slides were washed twice for 5 min in 170 mM Tris-HCl, 5 mM MgCl_2_ (pH 7.4) and 1% BSA and once for 5 min in 50 mM Tris-HCl with 5 mM MgCl_2_ (pH 7.4). Next, the slides were dipped in water, after which slides were dried using a heat gun. The activity on the tissue slides was measured using a Beaquant^®^—real time autoradiography machine (AI4R, Nantes France). To determine the amount of activity corresponding to the measured counts, 1 µL of treatment solution was pipetted onto a microscope slide, air dried and measured together with the samples. From the known molar activity of the treatment stock, the corresponding relative concentration (fmol/mm^2^) of the PSMA receptor could be calculated. The Kd and Bmax values for each tissue were calculated using a non-linear regression model in GraphPad Prism 9. The BP was calculated by dividing Bmax over Kd.

### 4.7. Cytotoxicity Assay of Monosodium Glutamate, Ionotropic and Metabotropic Glutamate Receptor Antagonists

The cytotoxicity of the different compounds under investigation was determined using the sulforhodamine B (SRB) based In vitro toxicology assay kit (TOX6, Sigma-Aldrich) as described below for the different compounds.

PC3-Flu and PIP cells were seeded at 10,000 cells/well, while A-253 cells were seeded at 15,000 cells/well in a 96-well plate. After overnight incubation at 37 °C and 5% CO_2_, cells were treated as follows.

To determine the cytotoxicity of MSG, the cells were incubated with increasing concentrations of MSG (0.9, 9, 90, 900 and 9000 µM) for 4 h. Next, the cells were washed twice and then fixed by adding 50% trichloroacetic acid (¼ of well volume) to the cell cultures. After 1 h of incubation at 4 °C, the wells were washed three times with water and air dried. Next, fixed cells were stained using 0.4% sulforhodamine B solution. After 30 min incubation at room temperature, cells were washed twice with 1% acetic acid wash solution and the incorporated dye was solubilized in 10 mM Tris. The absorbance was then measured using a CLARIOstar plate reader (BMG Labtech, Ortenberg, Germany). Cell survival was calculated relative to the untreated control condition and data were represented as a cell survival percentage. The cytotoxicity of ionotropic and metabotropic glutamate receptors was investigated as described above and the tested concentration for each compound is summarized in Table 3.

### 4.8. Blocking Studies Using Monosodium Glutamate, Ionotropic and Metabotropic Glutamate Receptor Antagonists on Cells

PC3-PIP cells were seeded at 10,000 cells/well, while A-253 cells were seeded at 15,000 cells/well. After overnight incubation, the cells were treated with 5 nM [^177^Lu]Lu-PSMA-617 and different concentrations of MSG and ionotropic and metabotropic glutamate receptor antagonists as summarized in Table 4. To compare the blocking capacity of the different tested compounds, the cells were also treated with 5 µM 2-PMPA. The treatment was incubated for 4 h at 37 °C with 5% CO_2_. Cells were then washed twice with PBS and the total bound fraction (membrane bound + internalized fraction) was collected by incubating the cells with 1 M NaOH for 30 min and washing them twice. Radioactivity in the collected samples was measured using a 2480 WIZARD^2^ automatic y-counter (Perkin-Elmer, Mechelen, Belgium). Data are represented as the relative binding percentage normalized to the untreated control condition.

### 4.9. Blocking Studies Using Monosodium Glutamate, Ionotropic and Metabotropic Glutamate Receptor Antagonists on Tissue

To validate the results found with the cellular blocking experiments, similar experiments were performed on mouse kidney and pig salivary gland tissue sections.

A similar protocol as described above for In vitro autoradiography was followed. Tissue sections were incubated with 5 nM [^177^Lu]Lu-PSMA-617, a vehicle (water or 2% DMSO) and different concentrations of the blocking compounds as summarized in Table 5. After a 1 h treatment of incubation and washing steps, sections were exposed using a Beaquant^®^—real time autoradiography machine (AI4R, Nantes, France). From the obtained counts per area, the relative binding percentage was calculated and normalized against the untreated vehicle condition. Data are represented as the relative binding percentage.

### 4.10. Immunohistochemistry

To characterize the different salivary glands sections, multiple tissue stainings were performed on paraffin-fixed tissues.

For all stainings, tissue slices were deparaffinized and rehydrated using xylol/ethanol and washed with demineralized water. For the hematoxylin and eosin staining, slices were then stained for 5 min with hematoxylin solution (105175, Merck millipore, MA, USA), washed and counterstained with Eosin Y-solution 0.5% aqueous (109844, Merck millipore, MA, USA). For the periodic acid—Schiff (PAS) staining, slides were incubated for 5 min in periodic acid 0.5% solution (100482, Merck millipore, MA, USA), washed and incubated for 15 min in Schiff’s reagent (3952016, Sigma-Aldrich, Hoeilaart, Belgium). Slides were then counterstained with hematoxylin. After staining and dehydration using ethanol/xylol, slides were mounted with a cover glass, dried and stored until imaging. Imaging was performed using a BioTek Cytation 5 (Agilent Technologies, Mechelen, Belgium)

### 4.11. Statistical Analysis

Tissue experiments were performed once in triplicate. Cell experiments were performed twice in triplicate. Tissue saturation binding experiments were analyzed using non-linear regression. Cytotoxicity data were analyzed using a 2-way ANOVA compared to the untreated control condition. Blocking studies were analyzed using a 2-way ANOVA with multiple comparisons and a Tukey post hoc test. For all experiments, outliers were detected and removed according to Tukey’s fences by the following formula: [Q1 − k(Q3 − Q1), Q3 + k(Q3 − Q1)], where k = 1.5. All statistical tests were performed using GraphPad Prism 9. Data were visualized as mean ± SD.

## 5. Conclusions

We investigated the uptake and retention of [^177^Lu]Lu-PSMA-617 in prostate (PC3-PIP) and salivary gland (A-253) cells as well as on tissue level using mouse kidney and pig salivary gland tissue. We showed low, non-specific binding in the salivary glands. This binding could be reduced by treatment with MSG, kynurenic acid and (RS)-MCPG. Further investigation is warranted to elucidate the exact mechanisms of salivary gland retention of PSMA-targeted small molecules.

## Figures and Tables

**Table 1:** Summary of the stability study of [^177^Lu]Lu-PSMA-617 at different time points after radiolabeling in different media.

	0 h	24 h	48 h	72 h
**Radiolabeling buffer**	98.7%	92.1%	85.0%	86.9%
**Phosphate-buffered saline**		94.3%	92.7%	92.7%
**Fetal bovine serum**		97.0%	96.0%	97.7%
**Complete growth media**		98.8%	97.9%	98.1%

**Table 2:** Summary of the binding affinity (Kd), PSMA receptor density (Bmax) and binding potential (BP).

	Kd (nM)	Bmax (fmol/mm^2^)	BP
**Mouse kidney**	2.8686	0.2356	4.1065
**Pig salivary gland 1**	4.6148	0.0029	0.0317
**Pig salivary gland 2**	146.6207	0.0178	0.0061
**Pig salivary gland 3**	8.6022	0.0059	0.0341

**Table 3:** Summary of the receptor class affinity and investigated concentrations of ionotropic and metabotropic glutamate receptor antagonists for the sulforhodamine B-based cytotoxicity assay.

Compound	Receptor Class Affinity	Investigated Treatment Concentrations (µM)
Ionotropic Glutamate Receptor Antagonists		
Kynurenic acid	NMDA, AMPA and kainate	0, 50, 500 and 1000
Memantine	NMDA	0, 50, 150 and 300
Capric acid	AMPA	0, 100, 500 and 1000
UBP 302	Kainate	0, 25, 100 and 250
MK 801 maleate	NMDA	0, 50, 200 and 500
**Metabotropic glutamate receptor antagonists**		
(RS)-MCPG	Group I + Group II	0, 10, 100, 500 and 1000
(RS)-CPPG	Group II + Group III	0, 50, 200, 500 and 1000
Ly341495	Group II	0, 10, 100 and 500
UBP1112	Group III	0, 10, 100 and 500

**Table 4:** Summary of the different compounds included in the cellular blocking studies and their concentrations used.

Compound	Investigated Treatment Concentrations (µM)
2-PMPA	5
Monosodium glutamate	0, 9, 900 and 9000
Kynurenic acid	0, 50, 500 and 1000
Memantine	0, 50, 150 and 300
Capric acid	0, 100, 500 and 1000
UBP 302	0, 25, 100 and 250
MK 801 maleate	0, 50, 200 and 500
(RS)-MCPG	0, 10, 100, 500 and 1000
(RS)-CPPG	0, 50, 200, 500 and 1000
Ly341495	0, 10, 100 and 500
UBP1112	0, 10, 100 and 500

**Table 5:** Summary of the different compounds and concentrations used in the tissue blocking studies.

Compound	Investigated Treatment Concentrations (µM)
2-PMPA	100
Monosodium glutamate	0, 9, 900 and 9000
Kynurenic acid	0 and 1000
Memantine	0 and 300
Capric acid	0 and 1000
UBP 302	0 and 250
MK 801 maleate	0 and 500
(RS)-MCPG	0 and 1000
(RS)-CPPG	0 and 1000
L341495	0 and 500
UBP1112	0 and 500

## Data Availability

Data is contained within the article.
